# Population pharmacokinetics of piperacillin/tazobactam in critically ill pediatric patients

**DOI:** 10.1128/aac.00272-26

**Published:** 2026-09-03

**Authors:** Tavey Dorofaeff, María Patricia Hernández-Mitre, Xin Liu, Yarmarly C. Guerra Valero, Mark G. Coulthard, Jeffrey Lipman, Steven C. Wallis, Diane L. Maresco-Pennisi, Suzane L. Parker, Jason A. Roberts

**Affiliations:** 1 Frazer Institute, The University of Queensland 1974, Brisbane, Australia; 2 Department of Pediatric Intensive Care, Queensland Children's Hospital 67568, Brisbane, Australia; 3 Faculty of Health Medicine and Behavioural Sciences, The University of Queensland 420004, Brisbane, Australia; 4 Jamieson Trauma Institute, Royal Brisbane & Women’s Hospital 3883, Brisbane, Australia; 5 Division of Anesthesia Critical Care, Pain and Emergency Medicine, UR-UM104 IMAGINE, University of Montpellier, Nimes University Hospital 27037, Montpellier, France; 6 Department of Intensive Care Medicine, Royal Brisbane & Women’s Hospital 550021, Brisbane, Australia; 7 Department of Pharmacy, Royal Brisbane & Women’s Hospital, Brisbane, Australia; 8 Herston Infectious Diseases Institute (HeIDI), Metro North Health 157827, Brisbane, Australia; University of Pennsylvania Perelman School of Medicine, Philadelphia, Pennsylvania, USA

**Keywords:** piperacillin-tazobactam, pharmacokinetics, critically ill children, pediatrics, intensive care

## Abstract

Piperacillin-tazobactam is commonly used in critically ill pediatric patients; however, standard dosing regimens may not reliably achieve maximally effective exposures in this population. To characterize the population pharmacokinetics of piperacillin-tazobactam in critically ill pediatric patients and evaluate the adequacy of commonly used dosing regimens, plasma concentrations were obtained around a single dose of piperacillin-tazobactam in critically ill pediatric patients aged 1 month to 12 years. A simultaneous population pharmacokinetic model for piperacillin and tazobactam was developed using nonlinear mixed-effects modeling. Monte Carlo simulations were performed to assess the probability of target attainment (PTA) for short, extended, and continuous infusion regimens across a range of ages and renal function states. Dosing adequacy was defined by simultaneous attainment of target drug exposures for piperacillin (100%fT > MIC) and tazobactam (85%fT > 2 mg/L). Thirty-one patients contributed 102 plasma samples, of which only 22.6% of patients achieved simultaneous piperacillin-tazobactam target exposures, considering a piperacillin MIC of 16 mg/L. Renal function and infusion duration were the primary determinants of attainment of target attainment. Standard short infusions frequently failed to achieve combined target exposures, particularly in patients with preserved renal function or augmented renal clearance (ARC). Extended infusions improved PTA, but remained suboptimal at higher MICs. Continuous infusion consistently achieved the highest target drug exposures across all simulated scenarios. Short intermittent piperacillin-tazobactam infusions and even extended infusions often result in suboptimal exposure in critically ill pediatric patients. Continuous infusion represents a rational dosing strategy to improve combined target attainment, particularly in children with preserved or ARC.

## INTRODUCTION

Piperacillin is a beta-lactam antibiotic (1) that is commonly combined with tazobactam (2, 3). Tazobactam is an irreversible beta-lactamase inhibitor that covalently binds to and inhibits bacterial beta-lactamase activity, thereby augmenting the clinical efficacy of piperacillin (4, 5). Piperacillin-tazobactam is commonly prescribed in critically ill pediatric patients with sepsis (6) because of its broad-spectrum activity against both gram-negative and gram-positive organisms, including *Escherichia coli*, *Pseudomonas*, *Staphylococcus*, *Streptococcus*, and *Enterococcus* species (7–9).

Previous studies evaluating piperacillin-tazobactam use in the pediatric intensive care unit (PICU) have raised concerns that therapeutic exposures are not consistently achieved (8–16), highlighting the need for studies to inform dose optimization strategies (13, 16–18). Although tazobactam is routinely co-administered with piperacillin, few studies have characterized its pharmacokinetics in critically ill children (15, 19, 20). Dose optimization is therefore more complex than for piperacillin alone, as maximally effective therapy requires simultaneous attainment of pharmacokinetic/pharmacodynamic (PK/PD) targets for both piperacillin and tazobactam (3–5).

In this study, we aimed to evaluate the appropriateness of commonly used piperacillin-tazobactam dosing regimens and to describe the population pharmacokinetics (PK) of both piperacillin and tazobactam in critically ill pediatric patients. This approach extends previous analyses focused on piperacillin by modeling both components of the combination and assessing combined target attainment for piperacillin and tazobactam. The final model was then used to perform dosing simulations to assess the likelihood of achieving maximally effective drug exposures for different dosing strategies across a range of ages and levels of renal function.

## RESULTS

### Patients

A total of 31 patients (11 infants and 20 children) were enrolled in the study, contributing 102 piperacillin and 97 tazobactam plasma concentrations for analysis (five tazobactam concentrations were below the lower limit of quantification and were excluded). The demographic and clinical characteristics of the study cohort are summarized in Table 1. More than half of the patients (61.3%) required mechanical ventilation. Based on estimated glomerular filtration rate (eGFR) categories, 19.4% of patients had an eGFR of 90–130 mL/min/1.73 m^2^), while 41.9% exhibited augmented renal clearance (ARC; defined as eGFR >130 mL/min/1.73 m^2^).

**TABLE 1:** Patient demographics and clinical characteristics^*a*^

Patients (*N* = 31)	Median (min–max)
Male, *n*/*N* (%)	14/31 (45.2%)
Age (years)	1.8 (0.1–11.8)
Age group, *n*/*N* (%)	
1 month–<1 year	11/31 (35.5%)
1–<4 years	8/31 (25.8%)
4–<9 years	6/31 (19.4%)
9–<12 years	6/31 (19.4%)
Height (cm)	84 (47–165)
Weight (kg)	10.80 (3–68)
PELOD-2	5 (0–12)
Mechanically ventilated, *n*/*N* (%)	19/31 (61.3%)
Vasopressors/inotropes, *n*/*N* (%)	12/31 (38.7%)
Albumin (g/L)	28 (19–48)
Serum creatinine (µmol/L)	28 (10–88)
eGFR Schwartz (mL/min/1.73 m^2^)	120.5 (54.3–289)
eGFR <90 mL/min/1.73 m^2^	10/29 (34.5%)
eGFR 90–130 mL/min/1.73 m^2^	6/29 (20.7%)
eGFR >130 mL/min/1.73 m^2^ (ARC)	13/29 (44.8%)
Reason for PICU admission	
Post-operative	15/31 (48.4%)
General/trauma	5/15 (33.3%)
Cardiac	5/15 (33.3%)
Neurosurgical	2/15 (13.3%)
Liver transplant	2/15 (13.3%)
Gastroenterology	1/15 (6.7%)
Laryngotracheal reconstruction	1/15 (6.7%)
Respiratory distress	4/31 (12.9%)
Congenital heart disease	4/31 (12.9%)
Altered level of consciousness	3/31 (9.7%)
Trauma	2/31 (6.5%)
Apnea	2/31 (6.5%)
Airway obstruction	1/31 (3.2%)
Heart failure	1/31 (3.2%)
Malignancy	1/31 (3.2%)
Paralysis tick bite	1/31 (3.2%)

^*a*^ PELOD-2, Pediatric Logistic Organ Dysfunction 2 score; eGFR, estimated glomerular filtration rate; ARC, augmented renal clearance; eGFR categories calculated in patients with available data; PICU, pediatric intensive care unit. Post-operative subcategories are reported among patients admitted post-operatively and are not mutually exclusive.

### Population PK modeling and validation

A one-compartment model with allometric scaling provided a superior description of the concentration-time data compared with two-compartment models for either compound, as evidenced by lower Akaike Information Criterion (AIC) values (Table S1). A combined constant–proportional error model best described residual unexplained variability for piperacillin, whereas a proportional error model was selected for tazobactam.

Inclusion of age and eGFR as covariates on clearance (CL) resulted in statistically significant reductions in objective function value (OFV) and an improved corrected Bayesian Information Criterion (BICc) (Table S1). These covariates explained 25.1% of interindividual variability (IIV) for piperacillin (16.16% attributable to age and 9.72% to eGFR) and 22.7% for tazobactam (14.78% attributable to age and 12.11% to eGFR) and were retained in the final population PK model. The final model equations were as follows:

### Piperacillin


$$CL_{i}\_pip=CL_{pop}\_pip\times {\left(\frac{WTi}{70}\right)}^{0.75}\times {\left(\frac{eGFRi}{117.9}\right)}^{0.76}\times {\left(\frac{AGEi}{1.8}\right)}^{0.2}$$

$$V_{i}\_pip=V_{pop}\_pip\times {\left(\frac{WTi}{70}\right)}^{1}$$

### Tazobactam


$$CL_{i}\_taz=CL_{pop}\_taz\times {\left(\frac{WTi}{70}\right)}^{0.75}\times {\left(\frac{eGFRi}{117.9}\right)}^{0.69}\times {\left(\frac{AGEi}{1.8}\right)}^{0.17}$$

$$V_{i}\_taz=V_{pop}\_taz\times {\left(\frac{WTi}{70}\right)}^{1}$$

where ${CL}_{i}$ and $V_{i}$ represent individual clearance and volume of distribution, ${WT}_{i}$ is body weight, ${eGFR}_{i}$ is estimated glomerular filtration rate, and ${AGE}_{i}$ is age for the ith individual, while ${CL}_{pop}$ and $V_{pop}$ represent the typical population estimates for clearance and volume of distribution.

**Fig 1:**
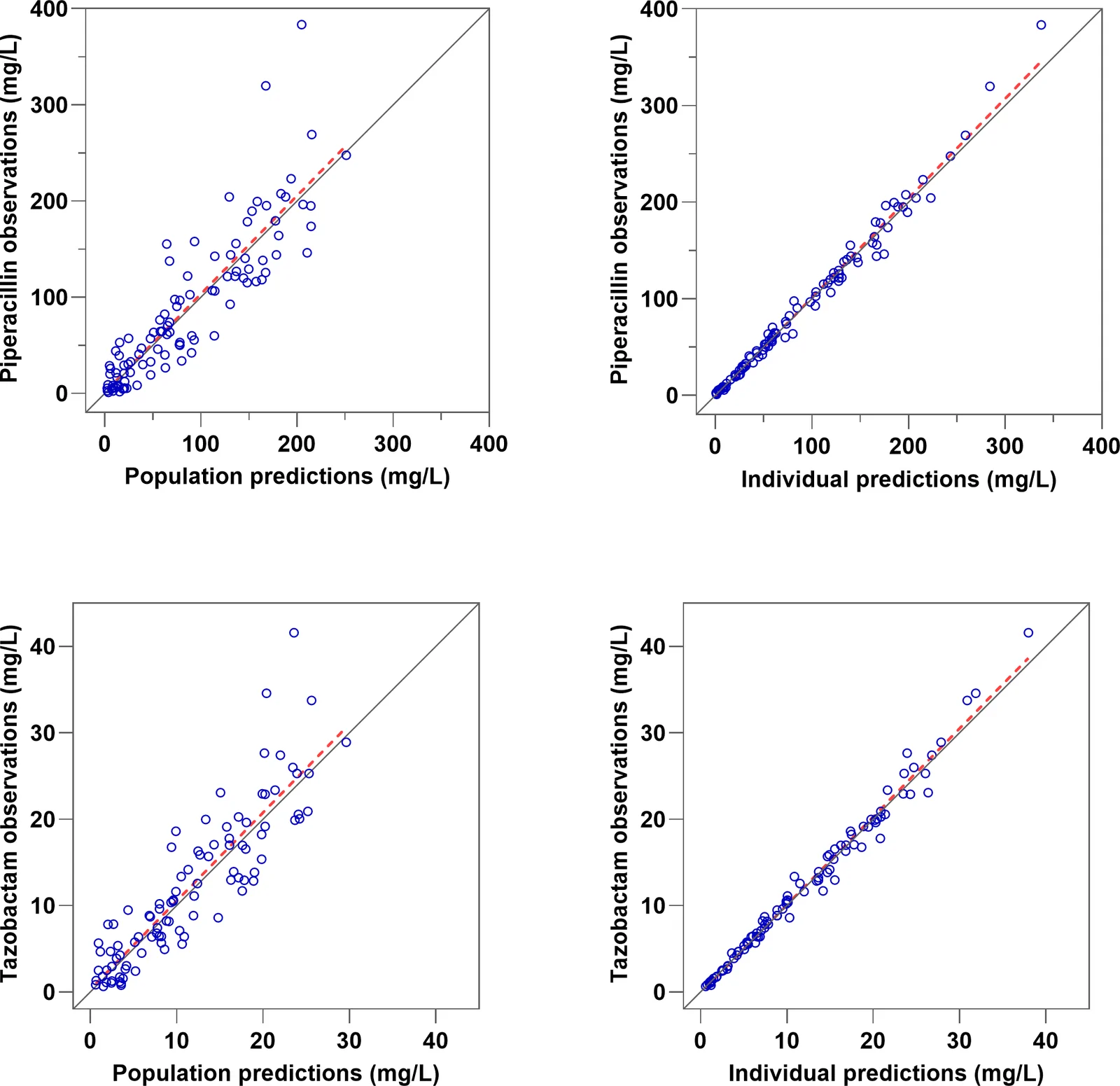
Goodness-of-fit plots for piperacillin (top) and tazobactam (bottom) of observations versus population predictions (left) and individual predictions (right).

**Fig 2:**
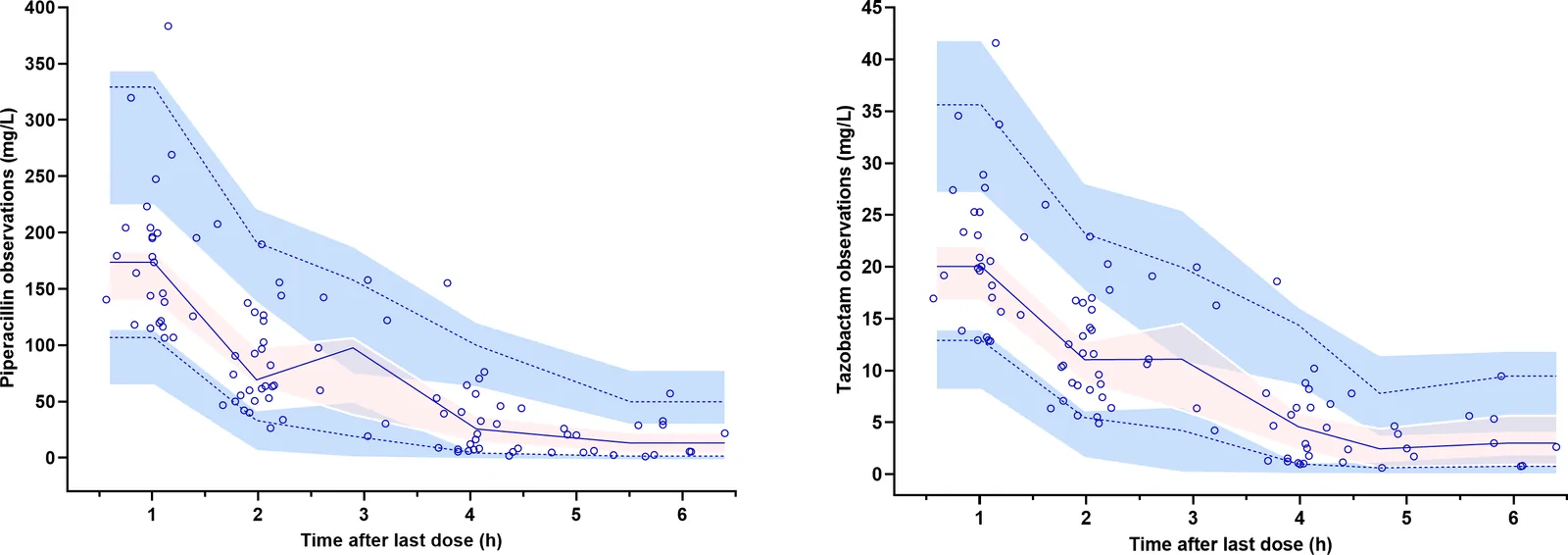
Visual predictive checks (VPCs) for piperacillin (left) and tazobactam (right). Dots represent observations; solid lines show the 5th, 50th, and 95th percentiles of the observations; shaded areas represent the 95% prediction intervals derived from simulations.

Final population PK parameter estimates are presented in Table 2. Goodness-of-fit (GOF) diagnostics demonstrated good agreement between observations and population and individual predictions for both compounds (Fig. 1). Additional diagnostic plots, including weighted individual residuals (IWRES) and normalized prediction distribution error (NPDE) plots, are provided in the Supplementary Material (Fig. S1 and S2). Visual predictive checks (VPCs) demonstrated that the final model adequately captured the central tendency and variability of the observed data for both piperacillin and tazobactam across the dosing interval (Fig. 2). Median bootstrap parameter estimates derived from 1,000 replicates, along with their corresponding 95% confidence intervals, were consistent with the final model estimates, supporting the robustness and stability of the final model.

**TABLE 2:** Piperacillin and tazobactam model parameters and bootstrapped values^*a*^

		Bootstrap (*N* = 1,000)
Parameter	Estimate (RSE%) [shrinkage, %]	Median (95% confidence interval)
Fixed effects		
CL_pip (L/h)	10.31 (5.35)	10.36 (9.28–11.4)
AGE_CL_pip	0.2 (18.3)	0.2 (0.12–0.27)
eGFR_CL_pip	0.65 (17.4)	0.65 (0.46–0.84)
V_pip (L)	24.9 (8.67)	25.01 (21.19–29.39)
CL_taz (L/h)	9.97 (5.15)	10 (8.94–10.99)
AGE_CL_taz	0.17 (19.6)	0.17 (0.095–0.25)
eGFR_CL_taz	0.69 (16.5)	0.69 (0.52–0.88)
V_taz (L)	28.61 (8.03)	28.53 (24.17–33.24)
Random effects, SD (ω)		
IIV_CL_pip	0.27 (15.4) [3.97] (CV 27.8%)	0.26 (0.17–0.34)
IIV_V_pip	0.44 (45.7) [3.83] (CV 46.0%)	0.42 (0.32–0.55)
IIV_CL_taz	0.27 (15.4) [3.99] (CV 27.3%)	0.26 (0.16–0.33)
IIV_V_taz	0.41 (34.3) [3.37] (CV 43.3%)	0.41 (0.29–0.55)
Correlations		
CL_pip and CL_taz	0.92 (6.93)	0.93 (0.83–0.96)
V_pip and V_taz	0.96 (18.4)	0.96 (0.93–0.97)
Residual unexplained variability		
Constant_pip	1.33 (31.1)	1.29 (0.78–1.85)
Proportional_pip	0.09 (15.9)	0.09 (0.06–0.12)
Proportional_taz	0.11 (10.0)	0.11 (0.08–0.14)

^*a*^ RSE, relative standard error; CI, confidence interval; CL_pip, piperacillin clearance; AGE_CL_pip, age effect on piperacillin clearance; eGFR, estimated glomerular filtration rate using the modified Schwartz equation; eGFR_CL_pip, eGFR effect on piperacillin clearance; V_pip, piperacillin volume of distribution; CL_taz, tazobactam clearance; AGE_CL_taz, age effect on tazobactam clearance; eGFR_CL_taz, eGFR effect on tazobactam clearance; V_taz, tazobactam volume of distribution; IIV, interindividual variability; random effects, including bootstrap medians and 95% confidence intervals, are reported as standard deviations of the random effects (ω).

Using individual Bayesian posterior predictions from the final population PK model, 35.5% (11/31) and 22.6% (7/31) of patients achieved simultaneous piperacillin and tazobactam target attainment at piperacillin minimum inhibitory concentrations (MICs) of 8 and 16 mg/L, respectively.

### Monte Carlo simulations and probability of target attainment

**Fig 3:**
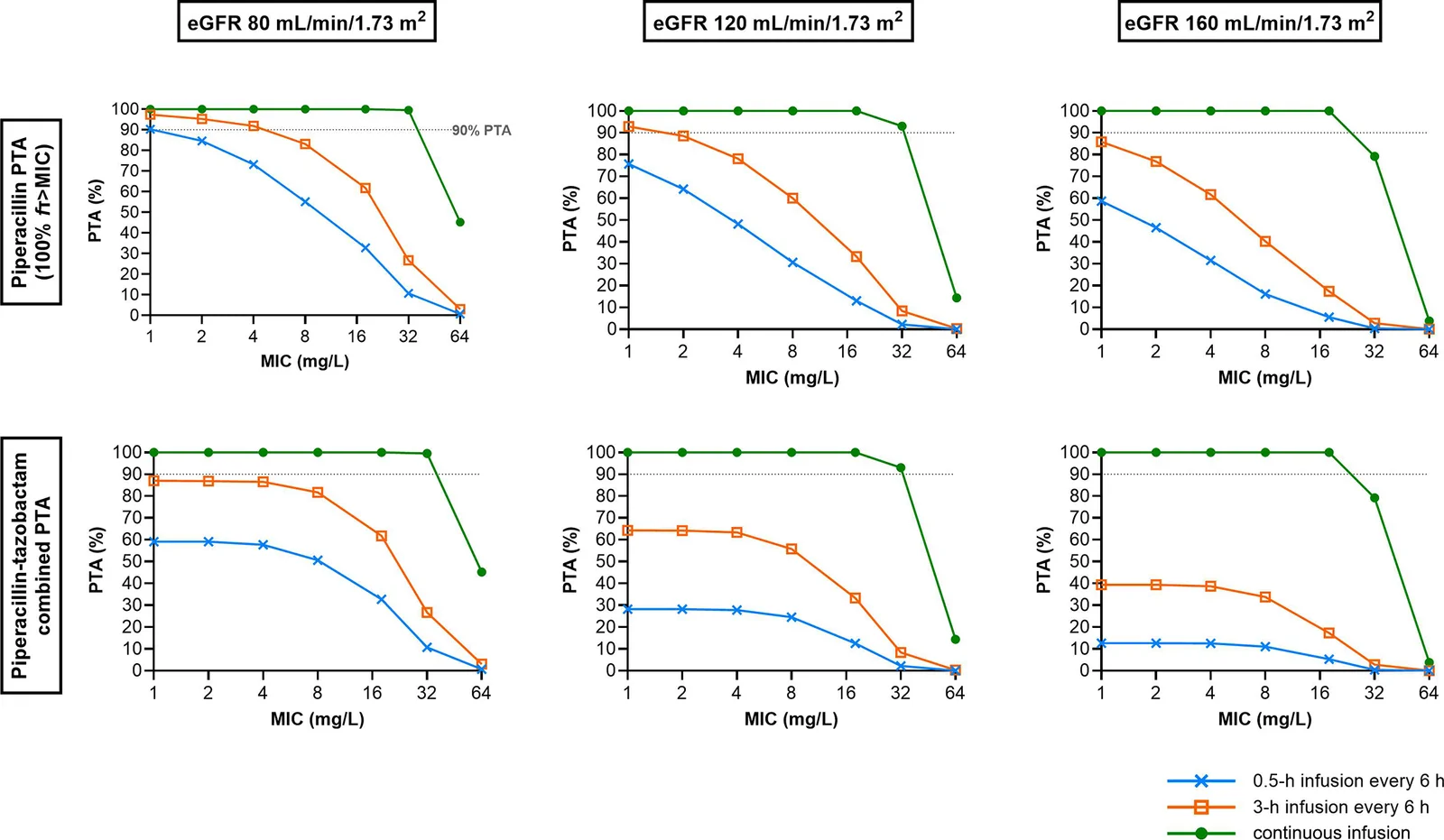
Probability of target attainment (PTA) for piperacillin (top panels) and combined piperacillin-tazobactam (bottom panels) across a range of minimum inhibitory concentrations (MICs) in a representative 2-year-old child weighing 11 kg. Panels are stratified by estimated glomerular filtration rate (eGFR) of 80, 120, and 160 mL/min/1.73 m^2^. Lines represent short intermittent 0.5-h infusion every 6 h (blue), extended 3-h infusion every 6 h (orange), and continuous infusion (green) of piperacillin 100 mg/kg with tazobactam 12.5 mg/kg. The dashed line indicates the 90% PTA threshold. In the top panels, PTA refers to attainment of the piperacillin target of 100% *f*T > MIC. In the bottom panels, PTA refers to simultaneous attainment of the piperacillin target of 100% *f*T > MIC and the tazobactam target of 85% *f*T > 2 mg/L.

Monte Carlo simulations demonstrated that the probability of target attainment (PTA) for piperacillin-tazobactam was strongly influenced by infusion duration and renal function across all age groups. Figure 3 presents PTA results for a representative 2-year-old child weighing 11 kg, illustrating the impact of infusion duration across a range of MICs and eGFR values.

As shown in Fig. 3, short intermittent infusion administered over 0.5 h resulted in a rapid decline in PTA with increasing MICs and frequently failed to achieve target drug exposures at MICs of 1–2 mg/L in patients with preserved renal function or ARC. Extended infusions administered over 3 h substantially improved PTA compared with short intermittent infusion; however, PTA decreased with increasing eGFR and higher MICs, with reduced target attainment observed in patients with ARC, particularly at MICs ≥4–8 mg/L. Continuous infusion consistently achieved the highest PTA across all simulated scenarios, maintaining high target attainment at MICs up to 8–16 mg/L in patients with eGFR values of 80–120 mL/min/1.73 m^2^ and outperforming short intermittent and extended infusion strategies in patients with ARC, especially at higher MICs.

Combined PTA for piperacillin-tazobactam followed similar trends to piperacillin alone and was frequently constrained by tazobactam exposure under short intermittent and extended infusion regimens. Overall, renal function and infusion duration were the primary determinants of PTA across the evaluated scenarios. PTA results for the representative 0.5- and 8-year-old scenarios are provided in the Supplementary Material (Fig. S3 and S4). These scenarios showed trends consistent with the 2-year-old reference scenario, with PTA decreasing as MIC increased and eGFR increased, improving with extended infusion, and being most consistently maintained with continuous infusion.

The additional age–weight scenario simulations confirmed the trends observed in the representative simulations. Across the evaluated scenarios, short intermittent infusion was associated with the lowest combined piperacillin-tazobactam PTA, extended infusion improved target attainment, and continuous infusion provided the most consistent PTA across renal function scenarios (Fig. S5).

Across all simulated scenarios, predicted trough concentrations remained below exposure thresholds previously associated with neurotoxicity.

## DISCUSSION

This study shows that dosing adequacy of piperacillin-tazobactam in critically ill pediatric patients is primarily governed by renal function and intravenous infusion duration. Our findings highlight that standard dosing regimens do not reliably account for variability in drug exposure in this population. These results underscore the clinical importance of considering alternative infusion strategies to mitigate the impact of ARC and to ensure adequate exposure to both components of the combination therapy.

Importantly, in this study, only a minority of patients achieved simultaneous piperacillin and tazobactam target drug exposures under standard dosing. This finding is consistent with a recent prospective PICU study, in which fewer than one-third of patients achieved beta-lactam target exposures, and an eGFR ≥90 mL/min/1.73 m^2^ was identified as a predictor of target non-attainment (21). Together, these findings emphasize ARC as a determinant of inadequate beta-lactam exposure in critically ill children.

Previous population PK studies in critically ill pediatric patients have consistently demonstrated substantial variability in piperacillin-tazobactam exposure and the frequent failure of standard short intermittent infusion regimens to achieve target drug exposures (8, 11, 19, 20). Across these analyses, body size, maturational factors, and renal function have consistently been identified as the principal determinants of CL, whereas markers of illness severity and supportive therapies have shown less consistent effects. These observations are consistent with the present findings and highlight the limitations of fixed dosing strategies in the PICU. Previous piperacillin-tazobactam population PK studies have reported both one-compartment (8, 20) and two-compartment (11) structural models. In this study, one-compartment structures were retained for both piperacillin and tazobactam based on model fit and precision of parameter estimates. The combined two-compartment model did not improve model fit and was associated with imprecision in peripheral volume parameter estimates, likely reflecting the sampling design and available data in the present study. Direct comparison of clearance and volume estimates across population PK studies is challenging because estimates are influenced by differences in patient population, sampling design, structural model, body size scaling, and renal function covariates.

A key strength of the present study is the simultaneous population PK modeling of both piperacillin and tazobactam in critically ill pediatric patients, coupled with the evaluation of combined PK/PD target attainment for both components. Although previous PICU studies have included tazobactam PK, dosing simulations and target attainment analyses have largely focused on piperacillin exposure alone. By incorporating a predefined tazobactam PK/PD target and assessing joint PTA, this analysis demonstrates that tazobactam may become a limiting factor under commonly used short and extended infusion regimens. This is clinically relevant, as piperacillin-tazobactam is prescribed as a fixed combination and therapeutic efficacy depends on adequate exposure to both agents. The present work therefore extends the PICU literature by providing a more complete assessment of combination therapy adequacy across a range of renal function states.

The superior performance of continuous infusion observed in this study is consistent with the time-dependent pharmacodynamics of beta-lactam antibiotics and prior clinical and PK studies demonstrating improved target attainment with continuous infusion (22–26). For piperacillin, maintaining free drug concentrations above the MIC for the entire dosing interval is critical for bacterial killing, and this becomes increasingly difficult to achieve in critically ill children with high renal clearance or ARC (8, 11, 13, 16). Continuous infusion minimizes fluctuations in plasma concentrations and reduces the impact of CL-driven variability, thereby increasing the likelihood of sustained target attainment across a broad range of renal function states (22, 24). Although loading doses were not evaluated in the present simulations, an initial loading dose may be useful when commencing continuous infusion to achieve target concentrations more rapidly, particularly in patients with high renal clearance. Importantly, this strategy also supports more consistent tazobactam exposure, which may otherwise become limiting under short intermittent or extended infusion regimens (3–5). From a clinical perspective, these findings support continuous infusion as a rational dosing strategy in critically ill pediatric patients at risk of subtherapeutic exposure, particularly those with preserved renal function or ARC. However, implementation of continuous infusion in pediatric patients can be challenging because of limited intravenous access, the need for line availability over prolonged periods, compatibility with other medications and fluids, and workflow considerations in the PICU. Where continuous infusion is not feasible, extended infusion may represent a more practical strategy to improve target attainment while reducing some of these implementation barriers.

This study has limitations that should be acknowledged. First, protein binding for both piperacillin and tazobactam was assumed rather than directly measured, which may introduce uncertainty into estimates of unbound exposure, particularly in critically ill children with altered plasma protein concentrations. Second, renal function was estimated using the modified Schwartz equation rather than measured directly, and while this reflects routine clinical practice, it may not fully capture dynamic changes in renal clearance in the PICU setting. In addition, ARC was defined in this study as eGFR >130 mL/min/1.73 m^2^; however, definitions of pediatric ARC vary across studies, including approaches based on creatinine clearance estimates or alternative thresholds (27). The sample size was modest; however, this reflects the inherent ethical and logistical challenges of conducting intensive PK studies in critically ill pediatric populations. Although the study included a clinically diverse cohort with a broad range of ages and renal function states, the small sample size limited the ability to evaluate a broader range of covariates and identify additional predictors of PK variability. Therefore, the covariate findings should be interpreted cautiously, and the generalizability of the model to broader PICU populations requires confirmation in larger external cohorts. Overall, 48.4% of patients were admitted to the PICU post-operatively (Table 1); however, details regarding intraoperative piperacillin-tazobactam administration, if any, and time from surgery to PK sampling were not available, limiting assessment of the potential influence of perioperative physiological changes on PK variability. In addition, clinical outcomes such as microbiological cure or mortality were not evaluated, precluding direct assessment of the relationship between target attainment and clinical efficacy. Finally, the population PK model has not yet been externally validated, and confirmation in larger, independent pediatric cohorts will be important to support broader generalizability of these findings.

### Conclusion

This study demonstrates that standard short intermittent infusions of piperacillin-tazobactam frequently fail to achieve optimal target drug exposures in critically ill pediatric patients, particularly in the presence of preserved renal function or ARC. Continuous infusion strategies were associated with more reliable target attainment across a range of renal function states and represent a rational dosing approach for critically ill children at risk of subtherapeutic exposure. Future multicenter studies incorporating clinical outcomes are needed to validate these findings and to inform evidence-based dosing strategies for piperacillin-tazobactam in the PICU.

## MATERIALS AND METHODS

### Study design

This prospective, open-label, observational study was conducted in the PICU at Queensland Children’s Hospital, Brisbane, Australia, between June 2019 and February 2023.

### Drug dosing and blood sampling

Initiation of therapy and dosing of piperacillin-tazobactam were at the discretion of the treating clinician. Piperacillin-tazobactam (AFT Pharmaceuticals) was administered every 6 h at a standard dose of 100/12.5 mg/kg (piperacillin/tazobactam) with a maximum of 4 g based on the piperacillin component. Doses were prepared from dry powder vials containing 4 g piperacillin and 0.5 g tazobactam, reconstituted with sterile water and further diluted to a standard concentration from which the prescribed dose was withdrawn. Piperacillin-tazobactam was administered intravenously over 30 min via a peripheral or central venous catheter, followed by a flush with 0.9% saline.

Blood samples were collected at five predefined time points: immediately prior to dosing (t1) and at 60 (t2), 120 (t3), 240 (t4), and 360 (t5) min following the start of the infusion. Actual sampling times were recorded and used in the population PK analysis. Samples were preferentially obtained from an indwelling arterial catheter when available or from venous catheters not used for antibiotic administration. Sampling from the antibiotic administration catheter was allowed only after adequate flushing. No additional catheters were inserted for blood sampling purposes.

### Bioanalytical assay

Total plasma concentrations of piperacillin and tazobactam were quantified using a validated ultra-high-performance liquid chromatography–tandem mass spectrometry (UHPLC–MS/MS) method on a Shimadzu Nexera2 UHPLC system coupled to a Shimadzu 8030+ triple quadrupole mass spectrometer (Shimadzu, Kyoto, Japan). The assay was conducted in accordance with the U.S. Food and Drug Administration (FDA) bioanalytical method validation guidance (28). The calibration range was 0.5–500 mg/L for total piperacillin and 0.625–62.5 mg/L for total tazobactam, with assay precision ≤7.8% and ≤10.8% and accuracy within 3.6% and 2.9%, respectively. The analytical method was adapted from a previously published and validated assay (29).

### Data collection

Patient demographic, biochemical, and clinical data were collected, including age, weight, sex, and renal function, defined by serum creatinine concentration and eGFR. eGFR was calculated using the modified Schwartz equation: eGFR (mL/min/1.73 m^2^) = 36.5 × height (cm)/serum creatinine (µmol/L). Markers of illness severity, including the Pediatric Logistic Organ Dysfunction 2 (PELOD-2) score, were recorded, along with the use of mechanical ventilation and vasopressors/inotropes.

### Population PK modeling and validation

Population PK modeling was performed using the nonlinear mixed-effects modeling software Monolix (version 2024R1; Lixoft SAS, a Simulation Plus company, Antony, France) using the stochastic approximation expectation–maximization (SAEM) algorithm. A log-normal distribution was assumed for individual PK parameters. IIV was described as $\theta_{j}=\theta_{p}\times exp\left(\eta_{j}\right)$, where $\theta_{j}$ is the estimated PK parameter for the jth individual, $\theta_{p}$ represents the typical population value, and $\eta_{j}$ is a random variable assumed to follow a normal distribution with mean zero and variance $\omega^{2}$.

Piperacillin and tazobactam were modeled simultaneously by fitting concentration-time data for both analytes within a single joint population PK model. Separate structural PK parameters and residual error models were estimated for each compound. Interindividual variability was estimated separately for each compound, and correlations between corresponding random effects were evaluated to account for within-patient correlation in piperacillin and tazobactam disposition. One- and two-compartment models with first-order elimination were evaluated. Allometric scaling was included *a priori*, with fixed allometric exponents of 0.75 and 1 for clearance and volume of distribution, respectively, and was standardized to a typical adult body weight of 70 kg. Constant, proportional, and combined constant–proportional error models were evaluated to describe residual unexplained variability (ε). Model selection was informed by the AIC, OFV, BICc, and visual inspection of GOF plots.

Starting from the base model, the effects of age, PELOD-2 score, albumin, serum creatinine, eGFR, mechanical ventilation, and vasopressors/inotropes use on piperacillin and tazobactam PK parameters were evaluated. Age, PELOD-2 score, albumin, serum creatinine, and eGFR were evaluated as continuous covariates using power functions. Mechanical ventilation and vasopressors/inotrope use were evaluated as categorical covariates. Covariate model building was performed using stepwise forward inclusion followed by backward elimination. During forward inclusion, a covariate was retained if it resulted in a reduction in OFV ≥3.84 (*P* < 0.05; χ^2^ distribution). During backward elimination, a covariate was retained if its removal resulted in an increase in OFV ≥10.83 (*P* < 0.001).

Model evaluation was based on GOF diagnostics, including observations versus population and individual predictions, IWRES versus time and individual predictions, and NPDE versus time and population predictions. A VPC was performed using 500 simulations of the final model. Internal model validation was conducted using a bootstrap procedure, in which 1,000 resampled data sets were generated in Monolix. Median parameter estimates and 95% confidence intervals from the bootstrap were compared with estimates obtained from the original data set.

Individual target attainment in the study cohort was assessed using individual Bayesian posterior predictions from the final population PK model at piperacillin MICs of 8 and 16 mg/L; these MICs were selected to reflect clinically relevant piperacillin-tazobactam MIC thresholds, including susceptible and higher-MIC gram-negative pathogens.

### Monte Carlo simulations and probability of target attainment

Monte Carlo simulations (*n* = 1,000 per scenario) were performed using Simulx (version 2024R1; Lixoft SAS, a Simulations Plus company, Antony, France), based on the final population PK model. Total plasma concentration-time profiles were simulated, and unbound concentrations were derived assuming a protein binding of 30% for both piperacillin and tazobactam. Representative simulations were conducted for patients aged 0.5, 2, and 8 years, with corresponding body weights of 6.5, 11, and 32 kg. To enable comparison across pediatric age groups, additional simulations were performed using clinically plausible age–weight scenarios, including 0.5 years/6.5 kg, 1 year/9 kg, 2 years/12 kg, 4 years/17 kg, 6 years/22 kg, 8 years/32 kg, 10 years/40 kg, and 12 years/50 kg. All representative and additional age–weight scenarios were evaluated at eGFR values of 80, 120, and 160 mL/min/1.73 m^2^. Simulated dosing regimens included piperacillin 100 mg/kg and tazobactam 12.5 mg/kg administered every 6 h, delivered as 0.5-h and 3-h intravenous infusions, as well as continuous infusion.

PTA was defined as the percentage of simulated individuals achieving the specified target drug exposures, which were defined to be maximally effective (30). For piperacillin, the target was defined as 100% of the dosing interval during which free (unbound) plasma concentrations remained above the MIC (100% *f*T > MIC) over an MIC range of 1–64 mg/L. MICs up to 64 mg/L were simulated to fully characterize the limits of target attainment under extreme exposure scenarios and to facilitate comparison with 4 × MIC targets. For tazobactam, the target was defined as 85% of the dosing interval during which free plasma concentrations exceeded 2 mg/L (85% *f*T > 2 mg/L). Combined piperacillin-tazobactam PTA was defined as the percentage of patients with simultaneous attainment of the piperacillin target (100% *f*T > MIC) and the tazobactam target (85% *f*T > 2 mg/L). Piperacillin trough concentration (Cmin) of 160 mg/L was used as a reference exposure threshold for beta-lactam-related neurotoxicity (31–33).

## SUPPLEMENTAL MATERIAL

Supplemental material aac.00272-26-s0001.docx Fig. S1 to S5; Table S1.

ASM does not own the copyrights to Supplemental Material that may be linked to, or accessed through, an article. The authors have granted ASM a non-exclusive, world-wide license to publish the Supplemental Material files. Please contact the corresponding author directly for reuse.
