# Supplementary material for: Population pharmacokinetics of piperacillin/tazobactam in critically ill pediatric patients
Source: Antimicrob Agents Chemother. 2026 Sep 3;70(10):e00272-26. doi: 10.1128/aac.00272-26 (PMC13689451; doi:10.1128/aac.00272-26)
Supplement: Supplementary file 1 [file aac.00272-26-s0001.docx]

**SUPPLEMENTARY MATERIAL**

**Figure S1.** Weighted individual residuals (IWRES; top panels) versus time (left) and individual predictions (right), and normalized prediction distribution error (NPDE; bottom panels) versus time (left) and population predictions (right) for **piperacillin**.

**Figure S2.** Weighted individual residuals (IWRES; top panels) versus time (left) and individual predictions (right), and normalized prediction distribution error (NPDE; bottom panels) versus time (left) and population predictions (right) for **tazobactam**.

**Figure S3.** Probability of target attainment (PTA) for piperacillin (top panels) and combined piperacillin-tazobactam (bottom panels) across a range of minimum inhibitory concentrations (MICs) in a representative 0.5-year-old child weighing 6.5 kg, stratified by estimated glomerular filtration rate (eGFR) of 80, 120, and 160 mL/min/1.73 m^2^. Infusion duration and dosing are as described in Figure 3. The dashed line indicates the 90% PTA threshold; PTA targets are as described in Figure 3.

**Figure S4.** Probability of target attainment (PTA) for piperacillin (top panels) and combined piperacillin-tazobactam (bottom panels) across a range of minimum inhibitory concentrations (MICs) in a representative 8-year-old child weighing 32 kg, stratified by estimated glomerular filtration rate (eGFR) of 80, 120, and 160 mL/min/1.73 m^2^. Infusion duration and dosing are as described in Figure 3. The dashed line indicates the 90% PTA threshold; PTA targets are as described in Figure 3.

**Figure S5**. Probability of target attainment (PTA) for combined piperacillin-tazobactam across a range of minimum inhibitory concentrations (MICs) and clinically plausible age–weight scenarios, stratified by estimated glomerular filtration rate (eGFR) of 80 (left panels), 120 (middle panels), and 160 (right panels) mL/min/1.73 m^2^. Panels are arranged by infusion strategy: short 0.5-h infusion (top panels), extended 3-h infusion (middle panels), and continuous infusion (bottom panels). Cell values represent PTA (%) rounded to the nearest whole percentage.

**Table S1**. Summary of structural and covariate model building steps

| **Step** | **Model evaluated** | **AIC** | **BICc** | **OFV** | **ΔOFV** | **Decision** |
| --- | --- | --- | --- | --- | --- | --- |
| Structural model | 1CMT pip/1CMT taz | 1396.23 | 1425.02 | 1374.23 | — | Retained as base structural model |
| Structural model | 2CMT pip/1CMT taz | 1400.89 | 1432.97 | 1376.89 | — | Did not improve fit |
| Structural model | 1CMT pip/2CMT taz | 1420.87 | 1454.81 | 1396.87 | — | Did not improve fit |
| Structural model | 2CMT pip/2CMT taz | 1446.18 | 1481.98 | 1422.18 | — | Did not improve fit |
| Forward inclusion | Add eGFR on CL_pip | — | 1417.6 | 1363.37 | -10.86 | Retained for further testing |
| Forward inclusion | Add eGFR on CL_taz | — | 1405.99 | 1348.33 | -15.04 | Retained for further testing |
| Forward inclusion | Add age on CL_pip | — | 1385 | 1323.91 | -24.42 | Retained for further testing |
| Forward inclusion | Add age on CL_taz | — | 1367.22 | 1302.69 | -21.22 | Retained for further testing |
| Forward inclusion | Add age on V_pip | — | 1365.01 | 1297.05 | -5.64 | Retained for further testing |
| Forward inclusion | Add age on V_taz | — | 1362.35 | 1290.96 | -6.09 | Retained for further testing. Complete model |
| Backward deletion | Remove eGFR on CL_pip | — | 1381.82 | 1313.87 | +22.91 | Retained in the final model |
| Backward deletion | Remove eGFR on CL_taz | — | 1386 | 1318.04 | +27.08 | Retained in the final model |
| Backward deletion | Remove age on CL_pip | — | 1383.2 | 1315.24 | +24.28 | Retained in the final model |
| Backward deletion | Remove age on CL_taz | — | 1380.42 | 1312.46 | +21.5 | Retained in the final model |
| Backward deletion | Remove age on V_pip | — | 1364.17 | 1296.21 | +5.25 | Removed from final model |
| Backward deletion | Remove age on V_taz | — | 1365.01 | 1297.05 | +6.09 | Removed from final model |
| 1CMT, one-compartment model; 2CMT, two-compartment model; AIC, Akaike information criterion; BICc, corrected Bayesian information criterion; OFV, objective function value; CL_pip, piperacillin clearance; CL_taz, tazobactam clearance; eGFR, estimated glomerular filtration rate; V_pip, piperacillin volume of distribution; V_taz, tazobactam volume of distribution. ΔOFV for forward inclusion is relative to the preceding model building step. ΔOFV for backward deletion is relative to the complete model. | | | | | | |
